# ANCA-associated vasculitis with thoracic spinal canal dural involvement and hypertrophic cranial pachymeningitis: A case report

**DOI:** 10.1097/MD.0000000000048479

**Published:** 2026-05-01

**Authors:** Xin Yin, Wei Zhou

**Affiliations:** a School of Clinical Medicine, Shandong Second Medical University, Weifang, China; b Department of Rheumatology, Liaocheng People’s Hospital, Liaocheng, Shandong Province, China.

**Keywords:** ANCA-associated vasculitis, hypertrophic cranial pachymeningitis, spinal canal dural space-occupying lesion

## Abstract

**Rationale:**

Anti-neutrophil cytoplasmic antibody-associated vasculitis (AAV) is a group of small-vessel necrotizing vasculitides with minimal immune complex deposition. Hypertrophic cranial pachymeningitis, a rare complication of AAV, is characterized by focal/diffuse dural thickening and fibrosis, causing neurological dysfunction such as headache and optic nerve injury. While AAV-associated hypertrophic cranial pachymeningitis is well documented, reports of concurrent spinal dural involvement remain scarce. This case highlights the rare co-occurrence of cranial and spinal dural involvement in AAV, providing new clinical evidence to expand the understanding of AAV-related dural manifestations and improve diagnostic awareness among clinicians.

**Patient concerns:**

A 57-year-old male presented with fever, headache, otalgia, and hearing loss, and was diagnosed with granulomatosis with polyangiitis based on positive anti-proteinase 3 antibodies (664.3), cytoplasmic anti-neutrophil cytoplasmic antibody (1:10). Symptoms resolved with glucocorticoids, disease-modifying antirheumatic drugs, and anti-infective therapy. He later developed recurrent headache and lumbodorsal pain.

**Diagnoses:**

Thoracic magnetic resonance imaging (MRI) revealed a T10 to 12 epidural lesion, which was surgically resected. Pathology confirmed epidural vasculitis with necrotic/fibrous tissue, granulation, and inflammatory cell infiltration. Methylprednisolone and cyclophosphamide relieved back pain, but headaches persisted. Cranial MRI showed dural thickening in the cerebellar, posterior fossa, and foramen magnum regions, consistent with pachymeningitis. Cerebrospinal fluid analysis showed elevated mononuclear cells (35.00 × 10^6^/L), nucleated cells (40.00 × 10^6^/L), and protein (1.49 g/L), with negative cultures. The final diagnosis was granulomatosis with polyangiitis complicated by concurrent cranial and spinal dural involvement.

**Interventions:**

Initial management included glucocorticoids, disease-modifying antirheumatic drugs, and anti-infective therapy, which resolved the patient’s fever, otalgia, and hearing loss. Surgical resection was performed for the T10 to 12 epidural lesion, followed by immunosuppressive therapy with methylprednisolone and cyclophosphamide. After adjustment of the treatment regimen, the patient’s headache improved.

**Outcomes:**

Initial therapy resolved the patient’s fever, otalgia, and hearing loss. Surgical resection combined with immunosuppressive therapy relieved lumbodorsal pain but failed to resolve headaches initially; cranial MRI confirmed pachymeningitis, and cerebrospinal fluid analysis indicated inflammatory changes. After adjustment of the treatment regimen, the patient’s headache improved, and he has remained clinically stable during follow-up.

**Lessons:**

This case demonstrates that AAV can involve both cranial and spinal dura mater. Clinicians should consider dural involvement in AAV patients presenting with lumbodorsal pain.

## 1. Introduction

ANCA (anti-neutrophil cytoplasmic antibodies) are a group of autoantibodies targeting cytoplasmic components of neutrophils. Their antibody targets are proteinase 3 (PR3) or myeloperoxidase (MPO), and the immune response mediated by them is one of the core pathogenesis mechanisms of ANCA-associated vasculitis (AAV).^[1]^ AAV is a group of small-vessel necrotizing vasculitides with little or no immune complex deposition. AAV includes granulomatosis with polyangiitis (GPA), microscopic polyangitis (MPA) and eosinophilic granulomatosis with polyangiitis.^[2]^ Hypertrophic cranial pachymeningitis (HCP) is a rare disease characterized by localized or diffuse thickening and fibrosis of the dura mater, leading to neurological dysfunction. Clinical manifestations include localized/diffuse headache, optic nerve damage, ataxia, and spinal cord dysfunction. HCP is divided into primary and secondary forms. Autoimmune diseases are a common cause of secondary HCP, among which AAV and immunoglobulin G subclass 4 (IgG4)-related diseases are more frequent.^[3]^ AAV mostly manifests as involvement of intracranial or peripheral organs, while cases concurrently complicated by thoracic spinal dural involvement and hypertrophic cranial pachymeningitis are extremely rare in reports. By comprehensively documenting the clinical diagnosis and treatment process of this rare case with multisite involvement, this study also supplement the possibility that ANCA-related vasculitis may involve a larger area of the dura mater, providing more practical experience for clinicians – this is the novel and unique aspect of this research.

## 2. Case report

**Figure 1.**
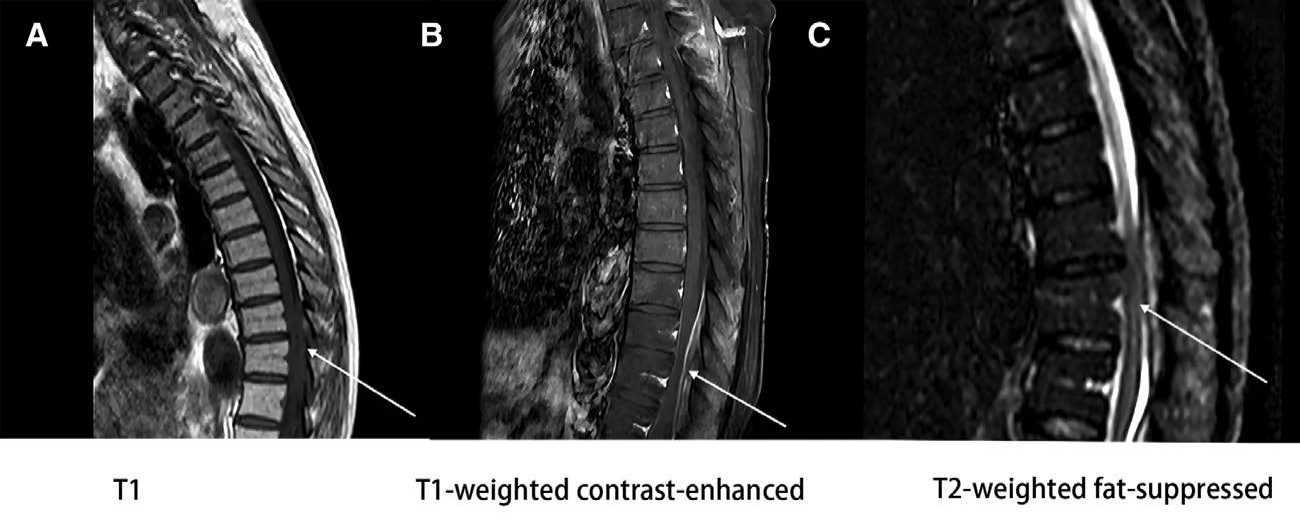
Enhanced MRI. (A–C) The area indicated by the arrow shows an abnormal signal in the epidural space at the posterior margin of the T10–12 vertebral canal. A thoracic spinal space-occupying lesion is considered. MRI = magnetic resonance imaging.

**Figure 2.**
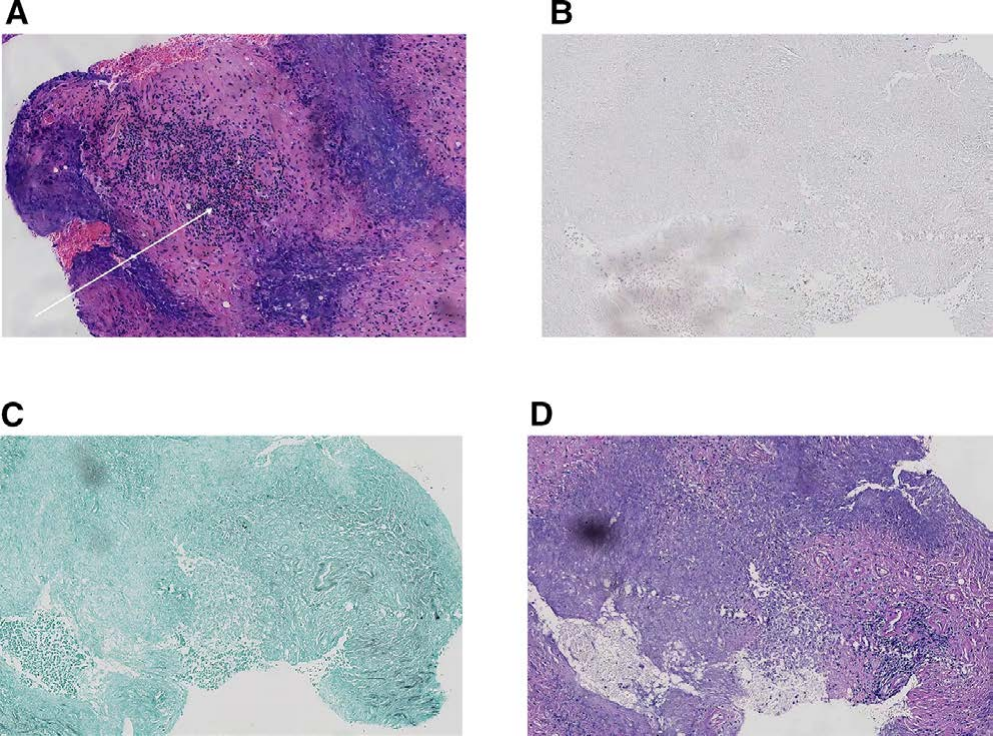
Pathological report (within the spinal canal). (A) The area indicated by the arrow shows proliferation of necrotic tissue, fibrous tissue, and granulation tissue, accompanied by calcification, as well as massive infiltration of acute and chronic inflammatory cells. Focal epithelioid cells and multinucleated cells are visible, which is consistent with inflammatory changes. (B) AFB (−). (C) GMS (−). (D) PAS (−). AFB = acid-fast bacillus, GMS = Gomori methenamine silver staining, PAS = periodic acid–Schiff reaction.

**Figure 3.**
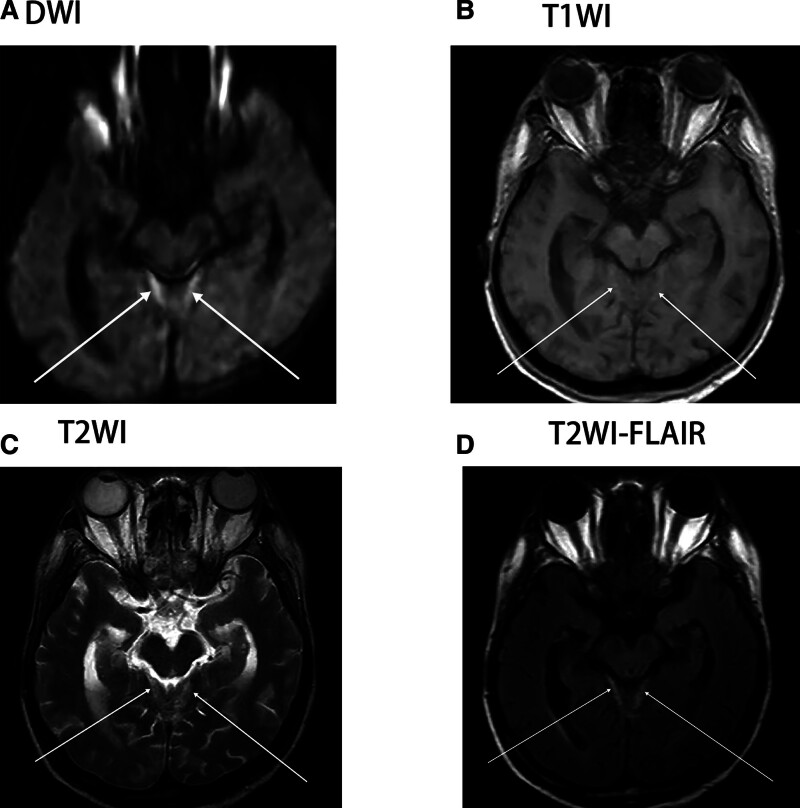
The MRI of the brain. (A–D) Symmetric slightly long T1 and slightly long T2 signals are observed in the area of the tentorium cerebelli near the midline (as indicated by the arrows), showing hyperintensity on FLAIR (fluid-attenuated inversion recovery) and diffusion restriction signal on DWI (diffusion-weighted imaging). DWI = diffusion-weighted imaging, FLAIR = fluid-attenuated inversion recovery, MRI = magnetic resonance imaging.

The patient, a 57-year-old male, was admitted to the ear, nose, and throat (ENT) Department of our hospital on October 11, 2023 due to “fever with headache, earache, nasal congestion, and hearing loss for 5 days.” After admission, the relevant auxiliary examinations suggested that *Pseudomonas aeruginosa* was present and sensitive to piperacillin, so symptomatic treatment such as piperacillin anti-infection and pain relief was given, and the patient’s headache and fever were slightly improved, and he was discharged on October 16, 2023, to Shandong Provincial Hospital for treatment. The vasculitis profile was completed: anti-proteinase 3 antibody (+), vasculitis antibody series: PR3 664.3, C-ANCA: 1:10 (+), the percentage of cluster of differentiation 3 (CD3) T cells: 79.03%, the percentage of cluster of differentiation 19 (CD19) B cells: 12.38%, abnormal liver function, while renal function, antiphospholipid antibody series, antinuclear antibody profile, autoimmune hepatitis antibody profile, tuberculosis infection T cell spot test (T-SPOT), G test, and Epstein–Barr virus (EB) virus showed no obvious abnormalities. The diagnosis of “granulomatosis with polyangiitis” was considered, and medications such as methylprednisolone 40 mg/d, mycophenolate mofetil 0.5 g bid, and ursodeoxycholic acid 250 mg tid were given. The patient was discharged after symptoms improved. After discharge, the glucocorticoid was gradually reduced to prednisone 7.5 mg/d. During this period, the patient complained of intermittent headache. He underwent a head MRI in our hospital in September 2024, which indicated multiple cerebral infarctions. The symptoms were not significantly relieved after symptomatic treatment. On November 11, 2024, the patient visited the Department of Spinal Surgery of our hospital due to “aggravated headache accompanied by low back pain for 1 month.” Enhanced MRI showed abnormal signal in the epidural space at the posterior edge of the T10–12 spinal canal. Considering thoracic spine occupancy (Fig. 1), intraspinal occupancy resection was performed on November 15, 2024. The pathological report of the resected lesion showed – intrathoracic spinal canal – necrotic tissue, fibrous tissue, and granulation tissue hyperplasia with calcification, a large number of acute and chronic inflammatory cell infiltration, and focal epithelioid cells and multinucleated cells, consistent with inflammatory changes. Special staining results: Gomori methenamine silver staining (GMS) (−), periodic acid–schiff reaction (PAS) (−), acid-fast bacillus (−) (Fig. 2). It is consistent with vasculitis combined with thoracic spine occupancy. The patient’s low back pain improved significantly after surgery. Due to the unsatisfactory relief of headache, brain MRI + DWI showed an abnormal signal in the area near the midline of the tentorium cerebelli (Fig. 3). The patient further visited Peking Union Medical College Hospital, where enhanced brain MRI + DWI was performed. The results revealed significant thickening of the dura mater in the bilateral cerebellum, posterior cranial fossa, and foramen magnum area, suggesting the possibility of pachymeningitis and given glucocorticoid shock (methylprednisolone 1 g/d for 3 days), intrathecal injection of methotrexate 10 mg, dexamethasone 10 mg, and combined rituximab treatment, he was discharged with relieved symptoms on March 14, 2025, The patient was followed up for 6 months, with sustained symptom remission and no signs of recurrence. For subsequent management, rechecks of ANCA and MRI are planned every 3 months (Table 1).

**Table 1:** Clinical course schedule.

Time node	Key events	Examination/treatment measures	Feedback on results
October 11, 2023	Fever accompanied by headache, earache, nasal congestion, and hearing loss	*Pseudomonas aeruginosa* infection is present, and is sensitive to piperacillin	Headache and fever improved slightly
October 16, 2023	Recurrent headache and fever	Vasculitis spectrum, vasculitis antibody series, cerebrospinal fluid (CSF) test	Anti-protease3 antibody (+), PR3 664.3, C-ANCA 1: 10(+), CSF examination:mononuclear cell count 35.00 × 10^6^/L, nucleated cell count 40.00 × 10^6^/L, protein quantification 1.49 g/L
November 2023–November 2024	Intermittent headache attacks	The MRI of the brain; methylprednisolone 40 mg/d, mycophenolate mofetil 0.5 g bid	The MRI of the brain showed multiple cerebral infarctions. After treatment, the condition still experienced recurrent episodes
November 11, 2024	New low back pain	Enhanced MRI; pathological examination	T10–12 posterior epidural extradural space signal. Consider epidural occupation. Pathological report: (within the spinal canal) necrotic tissue, fibrous tissue and granulation tissue hyperplasia, with caseation, massive acute inflammatory cell infiltration. Focal squamous epithelial cells and multiple Langhans giant cells were seen, consistent with granulomatous changes. Special staining results: GMS (−), PAS (−), acid-fast (−), consistent with angiitis complicated by epidural occupation
December 2024	Back pain after surgery relieved, but still has headache	Enhanced MRI of the brain and spinal cord	Bilateral cerebellum, posterior fossa, and large foramen magnum obvious thickening, possible pachymeningitis
December 2024–March 2025	After treatment, follow-up	Glucocorticoid shock (methylprednisolone 1 g/d for 3 d), intrathecal injection of methotrexate 10 mg, dexamethasone 10 mg, combined rituximab treatment	The condition is stable before menstruation, reexamination is scheduled every 3 mo

C-ANCA = cytoplasmic anti-neutrophil cytoplasmic antibody, CSF = cerebrospinal fluid, GMS = Gomori methenamine silver staining, MRI = magnetic resonance imaging, PAS = periodic acid−Schiff reaction, PR3 = anti-proteinase 3.

## 3. Discussion

Hypertrophic cranial pachymeningitis (HCP) is a rare inflammatory disease manifested by local or diffuse thickening of the dura mater of the cranial or spinal cord.^[4]^ The etiology of hypertrophic cranial pachymeningitis is diverse, mainly divided into idiopathic and secondary forms. Idiopathic pachymeningitis has no obvious cause and may be related to autoimmune abnormalities. The causes of secondary pachymeningitis are mainly divided into infectious and noninfectious. Infectious causes mainly include tuberculosis, syphilis, fungi, bacteria, etc, which can secondary pachymeningitis, causing inflammatory cell infiltration and dural thickening. Noninfectious etiologies mainly include systemic inflammatory diseases such as granulomatosis with polyangiitis, rheumatoid arthritis, systemic lupus erythematosus, mixed connective tissue disease, sarcoidosis, IgG4-related diseases, and tumor/traumatic diseases.^[5]^ In the differential diagnosis of this patient, infectious etiologies (such as tuberculous meningitis and fungal meningitis) were first ruled out: the patient’s cerebrospinal fluid (CSF) etiological tests (acid-fast staining, fungal culture) were all negative, so these were excluded. Secondly, neoplastic etiologies (such as subdural metastases and meningiomas) were ruled out: pathological examination showed necrotic tissue and granulation tissue proliferation (no tumor cells). Finally, combined with the positive ANCA result and the manifestation of multisite dural involvement, the patient was diagnosed with lesions caused by AAV. At present, the diagnosis of hypertrophic cranial pachymeningitis mainly relies on imaging. MRI examination and its enhanced scan are the preferred methods for diagnosing HCP, with the main manifestations: dural thickening, the thickened dura mater shows isointense/slightly hypointense signal on T1WI, significantly hypointense signal on T2WI, and significant enhancement on T1WI after enhancement. Coronal scan can show the “Mercedes-Benz” sign/“track” sign.^[6]^

Saggini et al further clarified that the mitogen-activated protein kinase (MAPK) pathway selectively regulates neutrophil chemotaxis and adhesion: activated extracellular signal-regulated kinase (ERK) upregulates neutrophil surface adhesion molecules to promote their transendothelial migration and dural infiltration, while dysregulated p38 MAPK disrupts macrophage polarization, worsening local proinflammatory microenvironments. These findings align with our patient’s cranial/spinal dural involvement and support MAPK inhibitors for refractory vasculitis.^[7]^ Avivar-Valderas (2023) noted that phosphatidylinositol 3-kinase (PI3Kβ)/mammalian target of rapamycin (mTOR) inhibition modulates immune responses. In our PR3-ANCA-positive case, ANCA-activated PI3Kβ/mTOR drives neutrophil hyperactivation, proinflammatory cytokine release, and dural vascular damage – consistent with the literature’s link between pathway dysfunction and immune dysregulation, confirming its pathological role in vasculitis. Given PI3Kβ/mTOR’s status as an immune regulatory target and our patient’s response to conventional therapy, inhibiting this pathway may further reduce vascular inflammation, offering a theoretical basis for targeted treatment of refractory vasculitis.^[8]^

A study showed that among patients with anti-neutrophil cytoplasmic AAV, 30 cases (4.52%) developed HCP, including 20 cases (3.58%) of newly diagnosed AAV patients and 10 cases (9.52%) of relapsed AAV patients. Among patients with HCP, 50% were classified as granulomatosis with polyangiitis (GPA). In all AAV patients and newly diagnosed AAV patients, the prevalence of GPA in patients with HCP was significantly higher than that in patients without HCP (*P* ≤ .001). In newly diagnosed AAV patients, the positive rate of serum proteinase 3 (PR3)-anti-neutrophil cytoplasmic antibody (ANCA) in patients with HCP was significantly higher than that in patients without HCP (*P* = .030), and the probability of ear, nose, and throat symptoms in patients with HCP was also significantly increased.^[9]^ Li et al reported 2 cases of AAV complicated with hypertrophic spinal pachymeningitis (HSP), and described the patients’ symptoms, examination results, and treatment conditions.^[10]^ This case documents the involvement of the thoracic spinal dura mater in AAV, and supplements the clinical value of multisite dural lesions, which forms a differentiated contribution compared with the cases reported by Li et al that only involved spinal meningitis.

Corticosteroids are the first-line treatment for HCP. Treatment is divided into 2 phases: induction and maintenance. Patients with relapse can manage symptoms with treatment with corticosteroids and DMARD, but it is important to note that infection must be strictly ruled out. For GPA induction, corticosteroids (methylprednisolone 1000 mg/d, pulse therapy for 3–5 days, then prednisone 1 mg/kg/d) combined with cyclophosphamide (initial dose 500–750 mg/m^2^ every 4 weeks, dose adjusted according to the lowest lymphocyte count, for 10–14 days) are used as first-line induction therapy.^[11]^ Studies have shown that PR3-AAV patients treated with rituximab (RTX) had a higher frequency of achieving complete remission (CR) at 6 months than those randomly treated with cyclophosphamide (CYC)/azathioprine (AZA; 65% vs 48%; *P* = .04). This suggests that rituximab can also be tried to induce disease remission in cases where cyclophosphamide induction therapy for refractory GPA fails.^[12]^ After remission, methotrexate and azathioprine can be maintained with therapy, and rituximab has become the best maintenance therapy drug, which can effectively reduce the recurrence rate.^[13]^ Relevant literature reports were searched through PubMed, Wanfang, and CNKI databases using keywords such as “ANCA, PR3, hypertrophic cranial pachymeningitis.” After layered screening, 23 articles from PubMed and 14 articles from CNKI were case reports consistent with ANCA-associated HCP, Nakajima et al conducted a retrospective study on hypertrophic pachymeningitis (HP). Among 61 patients with idiopathic/immune-mediated HP, only 6 cases had spinal dura involvement. The uniqueness of this medical record is that the patient with vasculitis not only involves the dura mater but also extends downward to involve the thoracic spinal dura.^[14]^ In this case, the patient presented with “atypical” symptoms such as low back pain and headache during the course of the disease, suggesting that the active stage of AAV can indicate disease progression or multisite involvement through new symptoms. It is necessary to timely complete imaging examinations (such as enhanced MRI) to clarify the involvement of rare sites such as the dura mater. The patient in this case had involvement of both the thoracic dura (spinal dura mater) and the cranial dura mater, confirming that the range of dural involvement in AAV is not limited to the intracranial dura mater, and the spinal dura mater can also be involved. Clinically, it is necessary to break the limited cognition that “dural involvement in vasculitis = dural mater involvement.” For patients with AAV, if they have spinal pain accompanied by neurological dysfunction (such as limb numbness, weakness) or persistent headache, the possibility of dural involvement should be vigilant, and enhanced MRI of the corresponding area should be completed as early as possible to confirm the diagnosis and avoid delaying treatment. As a case study focusing only on 1 specific subject, its conclusions are indeed difficult to be directly generalized to a broader population. We plan to collaborate with multiple centers to collect similar cases and further verify the phenomena observed in this case through small-sample pooled analysis or case-control studies, so as to gradually enhance the reference value of the conclusions.

## 4. Conclusion

There have been no previous reports on AAV complicated with thoracic dural involvement and hypertrophic cranial pachymeningitis. This case suggests that dural involvement in AAV is not limited to the cranial dura mater. When vasculitis patients have low back pain, the possibility of the primary disease involving the spinal canal dura should also be vigilant. It provides experience for the diagnosis and treatment of dural involvement in AAV and more basis for clinical practice.

## Author contributions

**Conceptualization:** Wei Zhou, Xin Yin.

**Data curation:** Wei Zhou.

**Formal analysis:** Xin Yin.

**Funding acquisition:** Wei Zhou.

**Investigation:** Wei Zhou.

**Methodology:** Wei Zhou.

**Project administration:** Wei Zhou.

**Resources:** Wei Zhou.

**Supervision:** Wei Zhou.

**Validation:** Wei Zhou, Xin Yin.

**Writing – original draft:** Xin Yin.

**Writing – review & editing:** Wei Zhou.
